# Integrated multiple-microarray analysis and mendelian randomization to identify novel targets involved in diabetic nephropathy

**DOI:** 10.3389/fendo.2023.1191768

**Published:** 2023-07-10

**Authors:** Chenyu Fan, Yuye Gao, Ying Sun

**Affiliations:** ^1^ Department of Cardiology and Institute of Vascular Medicine, Peking University Third Hospital, Beijing, China; ^2^ Key Laboratory of Carcinogenesis and Translational Research (Ministry of Education), Department of Gastrointestinal Surgery III, Peking University Cancer Hospital & Institute, Beijing, China; ^3^ Jiangsu Key Laboratory of New Drug Research and Clinical Pharmacy, Xuzhou Medical University, Xuzhou, Jiangsu, China

**Keywords:** diabetic nephropathy, microarray analysis, mendelian randomization, MICB, GZMA

## Abstract

**Background:**

Diabetic nephropathy (DN), which is the main cause of renal failure in end-stage renal disease, is becoming a common chronic renal disease worldwide. Mendelian randomization (MR) is a genetic tool that is widely used to minimize confounding and reverse causation when identifying the causal effects of complex traits. In this study, we conducted an integrated multiple microarray analysis and large-scale plasma proteome MR analysis to identify candidate biomarkers and evaluate the causal effects of prospective therapeutic targets in DN.

**Methods:**

Five DN gene expression datasets were selected from the Gene Expression Omnibus. The robust rank aggregation (RRA) method was used to integrate differentially expressed genes (DEGs) of glomerular samples between patients with DN and controls, followed by functional enrichment analysis. Protein quantitative trait loci were incorporated from seven different proteomic genome-wide association studies, and genetic association data on DN were obtained from FinnGen (3676 cases and 283,456 controls) for two-sample MR analysis. External validation and clinical correlation were also conducted.

**Results:**

A total of 82 DEGs (53 upregulated and 29 downregulated) were identified through RRA integrated analysis. The enriched Gene Ontology annotations and Kyoto Encyclopedia of Genes and Genomes pathways of the DEGs were significantly enriched in neutrophil degranulation, neutrophil activation, proteoglycan binding, collagen binding, secretory granule lumen, gluconeogenesis, tricarboxylic acid cycle, and pentose phosphate pathways. MR analysis revealed that the genetically predicted levels of MHC class I polypeptide-related sequence B (MICB), granzyme A (GZMA), cathepsin S (CTSS), chloride intracellular channel protein 5, and ficolin-1 (FCN1) were causally associated with DN risk. Expression validation and clinical correlation analysis showed that MICB, GZMA, FCN1, and insulin-like growth factor 1 may participate in the development of DN, and carbonic anhydrase 2 and lipoprotein lipase may play protective roles in patients with DN.

**Conclusion:**

Our integrated analysis identified novel biomarkers, including MICB and GZMA, which may help further understand the complicated mechanisms of DN and identify new target pathways for intervention.

## 1 Introduction

Diabetic nephropathy (DN) is a major microvascular complication of diabetes mellitus and the main cause of end-stage renal disease worldwide (1). DN is characterized clinically by decreased glomerular filtration rate (GFR) and increased serum creatinine and proteinuria (2) while exhibiting mesangial cell proliferation, hypertrophy, and expansion of the mesangial matrix at the cellular level (3). Metabolic factors, such as oxidative stress, elevated glucose levels, glomerular hypertension, and inflammatory chemokines, play key roles in the glomerular injury of renal cells and extracellular matrix deposition in DN (3, 4). However, the precise molecular mechanisms involved in the pathogenesis of DN have not yet been fully elucidated. Thus, further studies are needed to explore novel diagnostic targets and therapeutic strategies for DN.

Gene-specific expression profiling has recently been extensively adopted to analyze microarray data using bioinformatics methods (5, 6). Microarray technology has been widely used for gene expression patterns in renal tissues from patients with DN or experimental animals. However, some inconsistencies in those microarray studies have not been avoided or reduced, such as diverse microarray platforms, different sample sizes and data outliers, or even sources. However, in our study, the robust rank aggregation (RRA) method was utilized to combine and integrate the differentially expressed mRNA profiles of each of the selected datasets for high computational efficiency and statistical accuracy (7). Previous studies on the bioinformatic analysis of DN have not employed the RRA method to systematically incorporate differentially expressed genes (DEGs), which was facilitated this study.Mendelian randomization (MR) is an epidemiological approach that can detect the causal effect of exposure (e.g., plasma protein) on outcome (DN) using genetic variants as instrumental variables. Compared with observational studies, MR can avoid environmental confounders and reverse causality because the genetic variants used in MR cannot be easily changed by the external environment (8). Several genome-wide association studies (GWASs) of plasma proteins have recently identified the *cis*-variant in the protein-encoding gene (known as the protein quantitative trait loci, pQTL) for thousands of plasma proteins (9–15). Consequently, *cis*-pQTLs have been widely used as genetic instruments to estimate the causal effects of plasma proteins on complex diseases, satisfying three key assumptions of MR (relevance, independence, and exclusion assumptions) (16).

Thus, integrated multiple-microarray analysis was performed to identify DEGs in selected datasets using the RRA method, followed by gene enrichment and pathway annotation. We conducted a two-sample MR analysis of DEGs using *cis*-pQTLs extracted from seven different GWASs (9–15), followed by gene expression validation and clinical correlation analysis. With this, we aimed to identify candidate biomarkers and evaluate the causal effects of prospective therapeutic targets in DN, which will facilitate future mechanistic studies and drug discovery.

## 2 Materials and methods

### 2.1 Study design

**Figure 1:**
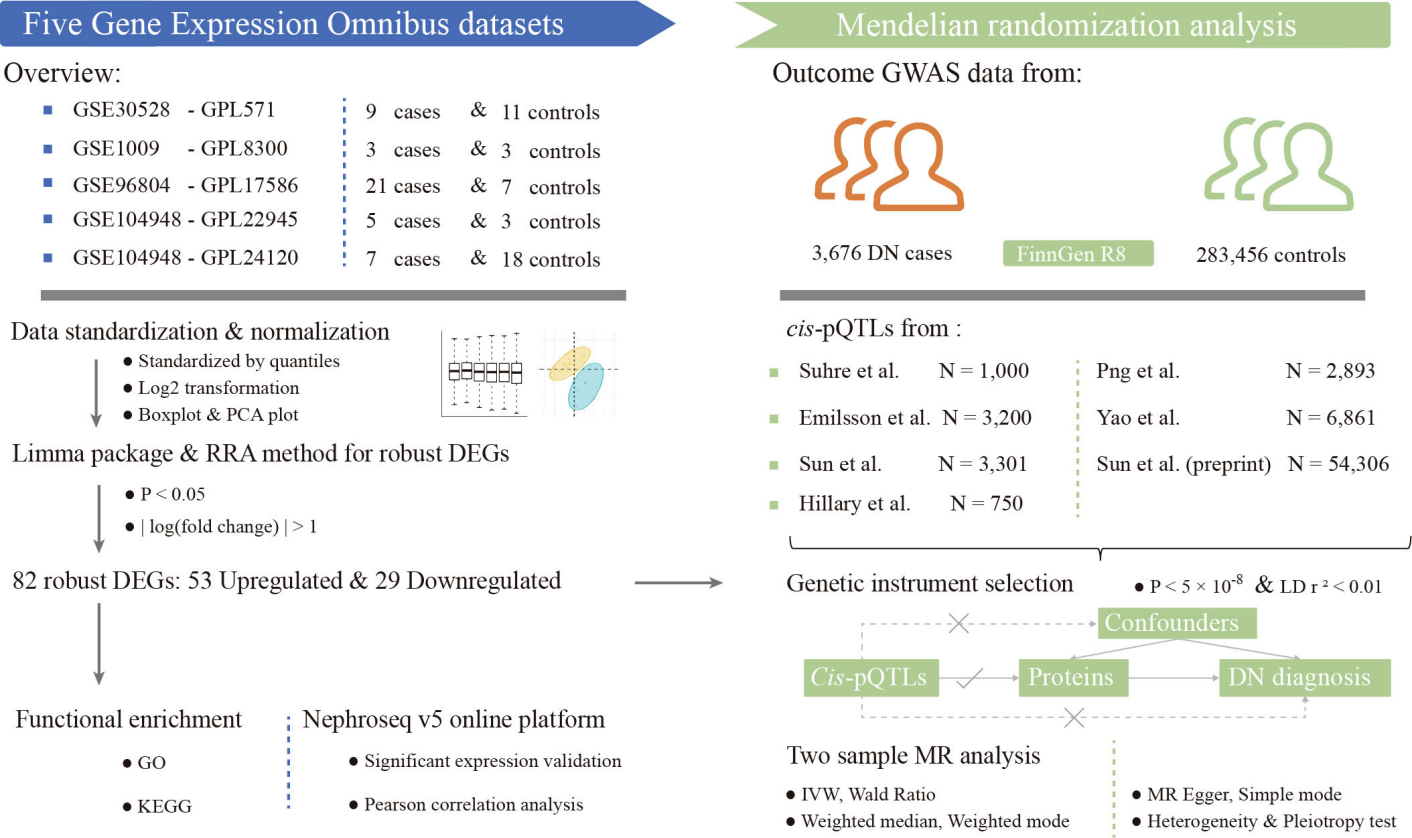
Study workflow. DEGs, differentially expressed genes; DN, diabetic nephropathy; GO, gene ontology; IVW, inverse-variance-weighted; KEGG, kyoto encyclopedia of genes and genomes; MR, mendelian randomization; pQTLs, protein quantitative trait loci; RRA, robust rank aggregation; GWAS, genome-wide association study.

**Figure 1** shows the study workflow. We first identified 82 DEGs from five GEO datasets using RRA methods. We then do the functional enrichment analysis to find out related pathological mechanisms. MR analysis was performed to elucidate causal inference for the association between DEGs encoded proteins and DN risk by using data from large-scale pQTLs studies. Expression validation and correlation with clinical parameters were conducted via the data in Nephroseq v5 online platform.

### 2.2 Microarray datasets of diabetic nephropathy

We obtained the gene expression datasets of diabetic nephropathy from the Gene Expression Omnibus (GEO) database (https://www.ncbi.nlm.nih.gov/geo/). We searched the GEO database by using the query terms: “Diabetic nephropathy”, “Glomeruli”, “Gene expression”, “Homo sapiens”, “Microarray”, and “mRNA”. Datasets were filtered under the following criteria (1): Containing at least 6 total samples; (2) Containing at least three cases and at least three controls; (3) Each sample in the dataset did not undergo any other chemical treatment or gene modification; (4) Raw data or gene expression profiling by array was available in the GEO datasets.

### 2.3 Data standardization and normalization

The gene expression matrix and related annotation files of each dataset were downloaded from the GEO database, and the gene symbols that the microarray probes correspond to were mapped for further analysis. The mean value was adopted if multiple probes were mapped to the same symbol. The datasets were then standardized by quantiles and the values of the genes that had undergone the log2 transformation. The boxplot indicating the overall expression of each sample in the five datasets was drawn by the graphics V4.0.2 package. For the further evaluation and verification of the key genes in terms of clearly distinguishing between diabetic nephropathy and healthy control samples, principal component analysis (PCA) was performed. For PCA, the prcomp function (https://stat.ethz.ch/R-manual/R-devel/library/stats/html/prcomp.html) was used to reduce the dimension of the data, and the PCA map was constructed by the factoextra V1.0.7 package.

### 2.4 RRA analysis

Differentially expressed genes (DEGs) with the threshold criterion of |logFC| >1 and p < 0.05 between diabetic nephropathy cases and healthy controls were screened by the limma V3.44.3 (Linear Models for Microarray and RNA-seq Data) package of the R software program (version 4.0.3). Then the ggplot2 V3.3.2 package was employed for the volcano plots of DEGs from each dataset. To reduce the inconsistencies to the minimum and to integrate the DEGs from five GSE datasets, Robust Rank Aggregation (RRA) method was employed to identify robust DEGs. Compared with the Venn plot, the RRA method is a more effective tool to integrate multiple microarray results (7). To perform the RRA analysis, we first calculated the up-ranked and down-ranked gene lists of each GEO dataset which were generated by expression fold change between diabetic nephropathy cases and healthy controls. The Robust Rank Aggregation V1.1 package was then used to integrate all the ranked gene lists of five GEO datasets. The adjusted *P-value* indicates the possibility of ranking high of each gene in the final results.

### 2.5 Functional and pathway enrichment analysis

Gene ontology (GO) and Kyoto Encyclopedia of Genes and Genomes (KEGG) analysis of differentially expressed genes in RRA analysis were performed using the clusterProfiler V3.16.1 package, which is a universal enrichment tool for functional and comparative study. P-value <0.05 and false discovery rate (FDR) < 0.05 was regarded as the cut-off criteria.

### 2.6 GWAS data source

We extracted pQTLs for plasma protein from seven different proteomic GWASs and integrated them using METAL (9–15, 17). Totally 72,331 cases and controls were included in our analysis. The ancestry of all of the individuals is European and there is no sample overlap among these seven data sources. Detailed information on the studies involved is listed in **Table S1**. Summary-level data on the association of DNA sequence variants with the DN risk were obtained from the FinnGen R8 study data release that contained 3676 cases and 283456 controls for the discovery cohort (18). Detailed information containing case definition and covariates are presented in **Table S1**.

All of the GWAS summary statistics employed in this study are publicly available and can be freely downloaded. Ethics approval was obtained by the original analysis.

### 2.7 Instrumental variable selection

As for genetic instrument selection, we screened pQTLs associated with proteins that were encoded by the robust DEGs previously identified by the RRA method and three key assumptions must be met (19). To meet assumption 1 (relevance assumption), we restricted the SNPs to be directly associated with the exposure at the P < 5 ×10^-8^ (genome-wide significant level), on the other hand, the F statistic > 10 was regarded as a good strength of the genetic instrument. Assumption 2 (independence assumption) is that the genetic variant should not be directly related to the confounders, which can be evaluated by the horizontal pleiotropy in the post-MR analysis.

The third MR assumption, known as the exclusion restriction assumption, means that the instrumental variables (IVs) should be associated with the outcome only via exposure. To meet this assumption, we elected to use only cis-acting SNPs (located only within 1 Mb of the genes that encode the proteins) (20) as IVs in our MR analysis and restrict the linkage disequilibrium (LD) clumped r2 < 0.01. Because *cis*-pQTLs are regarded to influence the protein definitely and directly compared with *tran*s-pQTLs, they are rarely likely to affect the levels of the protein independently of the levels of the proteins encoded by their corresponding genes. Instrument variables are listed in **Table S2**.

### 2.8 MR statistical analysis

After selecting eligible IVs and clumping with LD r^2^ < 0.01, most of the pQTLs have at most 2 eligible IVs. Next, the IVs in exposure GWAS were harmonized with that in outcome GWAS data., where the palindromic SNPs with intermediate allele frequency were removed. Moreover, the missing SNP was replaced by a proxy SNP with strong linkage disequilibrium (r2 ≥ 0.8). The Wald ratio was adopted in single IV MR and the inverse-variance-weighted (IVW) method was calculated for 2 SNPs or more. In addition, Egger’s regression and the weighted median were also conducted as references if applicable. The leave-one-out sensitivity analysis was performed to determine if a single SNP has a dramatic effect on the association between exposure of interest and the DN outcome. We also applied the MR-PRESSO method (the replicates were set 5000 times) to detect the outliers (21). The false discovery rate (FDR) was adopted to adjust the multiple comparisons. The steps above were performed using the “TwoSampleMR” R package (github.com/MRCIEU/TwoSampleMR) (22) and R software 4.2.2.

### 2.9 External validation and clinical correlation

For the validation of the targets we identified, we then used the data in Nephroseq v5 online platform (http://v5.nephroseq.org) to verify the significant expression of the target genes and the Pearson correlation analysis between serum protein expression and glomerular filtration rate (GFR) level, serum creatinine, proteinuria from samples of the patients with diabetic nephropathy. The query settings were set as follows: organism = homo sapiens, disease = diabetic nephropathy. Comparisons between the two groups were evaluated by using the unpaired Student t-test. The two-tailed P-value <0.05 was set as the screening criteria.

## 3 Results

### 3.1 Overview of five included GEO datasets

Five datasets were included in our study according to the defined criteria in method. **Table 1** provides detailed information on the included datasets. A total of 45 patients with DN and 52 healthy controls were included in these five datasets. The expression value of each gene in the dataset to which it belonged was standardized and normalized. The boxplots in **Figure S1** show that all the samples in each dataset achieved acceptable homogeneity. Principal component analysis plots of each dataset were obtained to reveal the distinct gene expression patterns between patients with DN and healthy controls (**Figure S2**). The samples from the DN cases were obviously separated from the normal samples of the healthy controls in each of the five GSE datasets, which promises a lower deviation and inconsistency for the following analysis.

**Table 1:** Characteristics of the five included GEO datasets.

GSE ID	Samples	Tissues	Analysis type	Platform
GSE30528	9 cases and 11 controls	kidney glomerulus	Array	GPL571
GSE1009	3 cases and 3 controls	kidney glomerulus	Array	GPL8300
GSE96804	21 cases and 17 controls	kidney glomerulus	Array	GPL17586
GSE104948	5 cases and 3 controls	kidney glomerulus	Array	GPL24120
GSE104948	7 cases and 18 controls	kidney glomerulus	Array	GPL22945

### 3.2 DEGs identification by RRA integrated analysis

**Figure 2:**
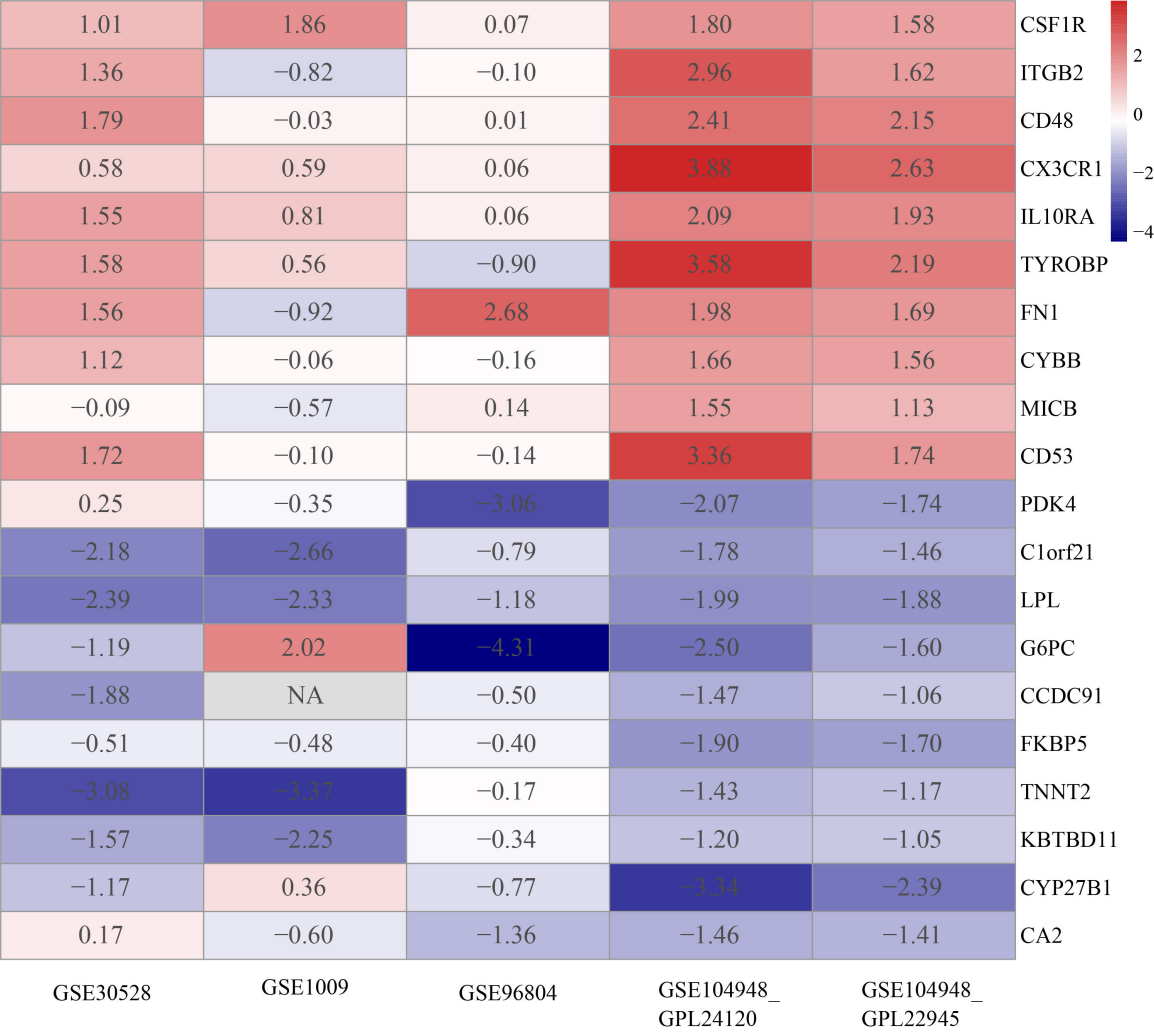
Heatmap of top 10 upregulated and top 10 downregulated DEGs. The red band represents the high expression of genes in diabetic nephropathy and the blue band represents the low expression of genes in diabetic nephropathy.

The DEGs of each GEO dataset were screened out using the limma package in R software according to the previously established criteria (|logFC| >1 and p < 0.05). The volcano plots of five GEO datasets were shown in **Figure S3**. In the RRA results, the smaller the p-value the higher gene ranks and the credibility of gene differential expression, and the significance scores provide a rigorous way to keep the statistically relevant genes. Through the RRA methods, 53 upregulated DEGs and 29downregulated DEGs were determined and the full results were in **Table S3**. The heatmap in **Figure 2** showed the top 10 upregulated DEGs and the top 10 downregulated DEGs.

### 3.3 GO and KEGG enrichment analysis

**Figure 3:**
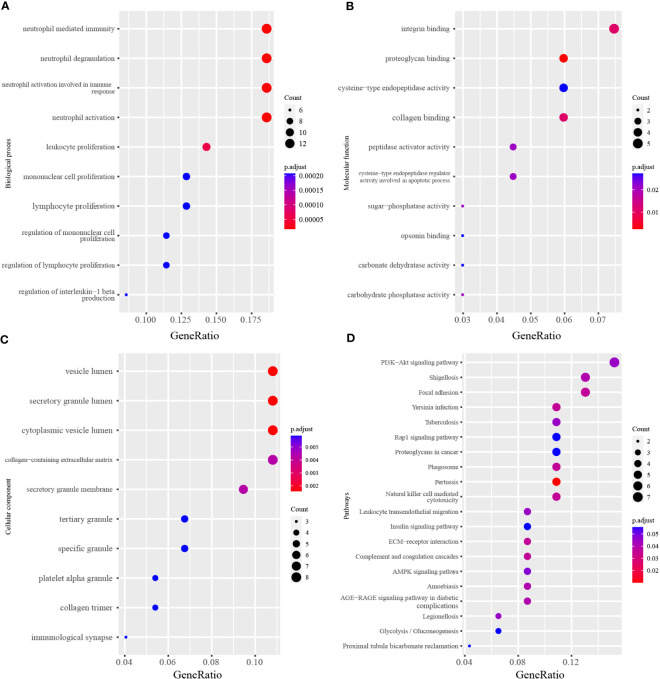
The GO and KEGG analysis of differentially expressed genes. **(A)** Top 10 terms of Biological process in GO analysis. **(B)** Top 10 terms of Molecular function in GO analysis. **(C)** Top 10 terms of Cellular component in GO analysis. **(D)** Top 20 terms of KEGG enrichment analysis. The x-axis label represents the gene ratio and the y-axis label represents the enriched terms.

The GO (including biological process, molecular function and cellular component) and KEGG enrichment were performed by the clusterProfiler package followed by the criteria previously mentioned. The results showed that neutrophil degranulation (GO:0043312; P-value = 2.57E-08), neutrophil activation involved in immune response (GO:0002283; P-value = 2.77E-08), neutrophil activation (GO:0042119; P-value = 3.51E-08), neutrophil mediated immunity (GO:0002446; P-value = 3.59E-08), leukocyte proliferation (GO:0070661; P-value = 1.57E-07) were the top 5 significantly enriched in biological process, followed by lymphocyte proliferation (GO:0046651; P-value = 7.75E-07), mononuclear cell proliferation (GO:0032943; P-value = 8.24E-07) and so on (**Figure 3A**). In terms of the molecular function, proteoglycan binding (GO:0043394; P-value = 1.01E-05) and collagen binding (GO:0005518; P-value = 1.20E-04) were the top 2 siginificantly ranked (**Figure 3B**). The secretory granule lumen (GO:0034774; P-value = 2.68E-05) was the most significantly enriched in the cellular component (**Figure 3C**). As for KEGG enrichment analysis, **Figure 3D** showed that Glycolysis/Gluconeogenesis (hsa00010; P-value = 3.69E-03), Steroid biosynthesis (hsa00100; P-value = 1.01E-01), Citrate cycle (TCA cycle) (hsa00020; P-value = 1.48E-01), Pentose phosphate pathway (hsa00030; P-value = 1.48E-01) were significantly enriched in KEGG analysis. The full results were available in **Table S4**.

### 3.4 MR analysis

**Figure 4:**
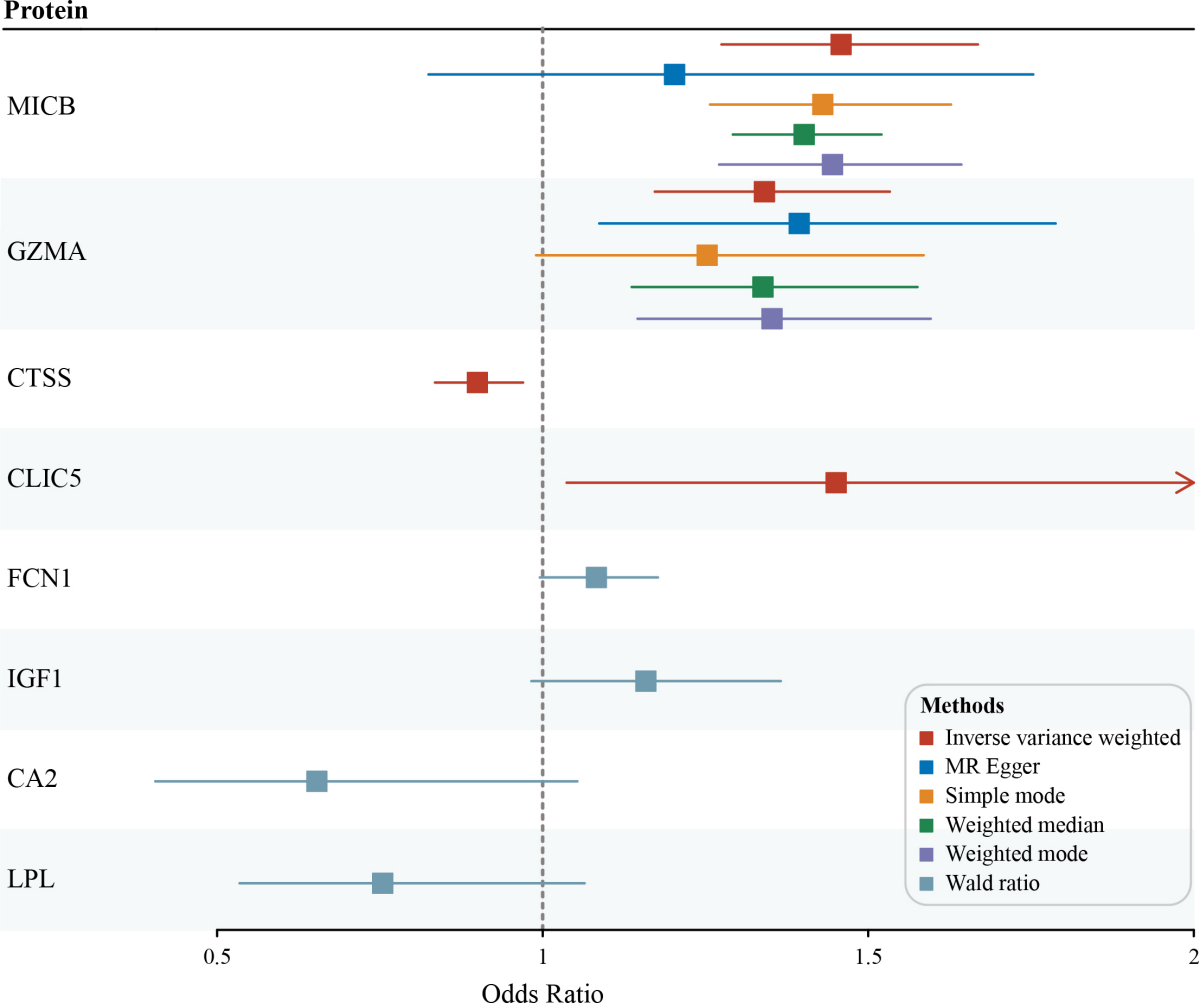
Potential casual associations of circulating proteins with risk of diabetic nephropathy (DN) in FinnGen outcome GWAS cohort.

Among the 82 identified DEGs associated proteins at the nominal significance level in DN, 57 were removed from MR analysis for lack of genetic instruments. The F statistics for all selected SNPs were over 10 (**Table S2**). A total of four different circulating plasma proteins showed causal effects on DN in the FinnGen cohort. As shown in **Figure 4**, higher genetically predicted levels of MICB (MHC class I polypeptide-related sequence B), GZMA (Granzyme A) and CLIC5 (Chloride intracellular channel protein 5) were associated with an increased risk of DN. Moreover, MICB and GZMA were both upregulated on genetic levels in the DN group according to our previous RRA integrated analysis, which further provided supporting evidence for their potentially casual association with an increased risk of DN. To detail, in the MR analysis using the inverse variance weighted method, the odd ratio of DN per standard deviation increase in genetically predicted levels of proteins was 1.46 (95% CI 1.27-1.67; *P* = 3.94 × 10^-8^) for MICB, 1.34 (95% CI 1.17-1.53; *P* = 1.86 × 10^-5^) for GZMA, 0.90 (95% CI 0.83-0.97; *P* = 5.78 × 10^-3^) for CTSS, and 1.45 (95% CI 1.04-2.03; *P* = 2.99 × 10^-2^) for CLIC5. Since there were 8 and 3 pQTLs identified for MICB and GZMA respectively, MR-Egger, simple mode, weighted median, and weighted mode were also performed. Except for MR-Egger in MICB and simple mode in GZMA, other methods mentioned above showed significant results for MICB and GZMA and all methods provided the same direction for the increased risk of DN (OR > 1). The Wald ratio analysis was conducted if only a single pQTL was identified for the protein. Combined MR analysis with the DEGs identification result, FCN1 (Ficolin-1) was upregulated on genetic levels and associated with a high risk of DN (OR = 1.08; 95% CI 1.00-1.18), IGF1 (Insulin-like growth factor I) was downregulated and linked with a high risk of DN (OR = 1.16; 95% CI 0.98-1.37), CA2 (Carbonic anhydrase 2; OR = 0.65; 95% CI 0.41-1.05) and LPL (Lipoprotein lipase; OR = 0.75; 95% CI 0.53-1.06) was downregulated and unfold low risk of DN, although these four proteins were not significant enough for lack of pQTLs in MR result. Sensitive analysis of identified proteins with DN is presented in **Table 2** and the MR associations for all studied proteins can be found in **Table S5**.

**Table 2:** Sensitive analysis of identified proteins with DN.

Protein	Method	nSNP	Pleiotropy				Heterogeneity	
			MR-PRESSO Global P-value	Egger intercept	Intercept’s se	MR Egger P-value	Q-value	P-value
MICB	IVW	5	0.718	0.079	0.070	0.341	2.973	0.562
	MR-Egger						1.696	0.638
GZMA	IVW	3	NA	-0.013	0.036	0.777	0.425	0.809
	MR-Egger						0.291	0.589
CTSS	IVW	2	NA	NA	NA	NA	0.022	0.883
CLIC5	IVW	2	NA	NA	NA	NA	0.008	0.929

NA, Not Applicable; nSNP, number of SNPs; IVW, Inverse variance weighted.

### 3.5 External validation and clinical correlation

**Figure 5:**
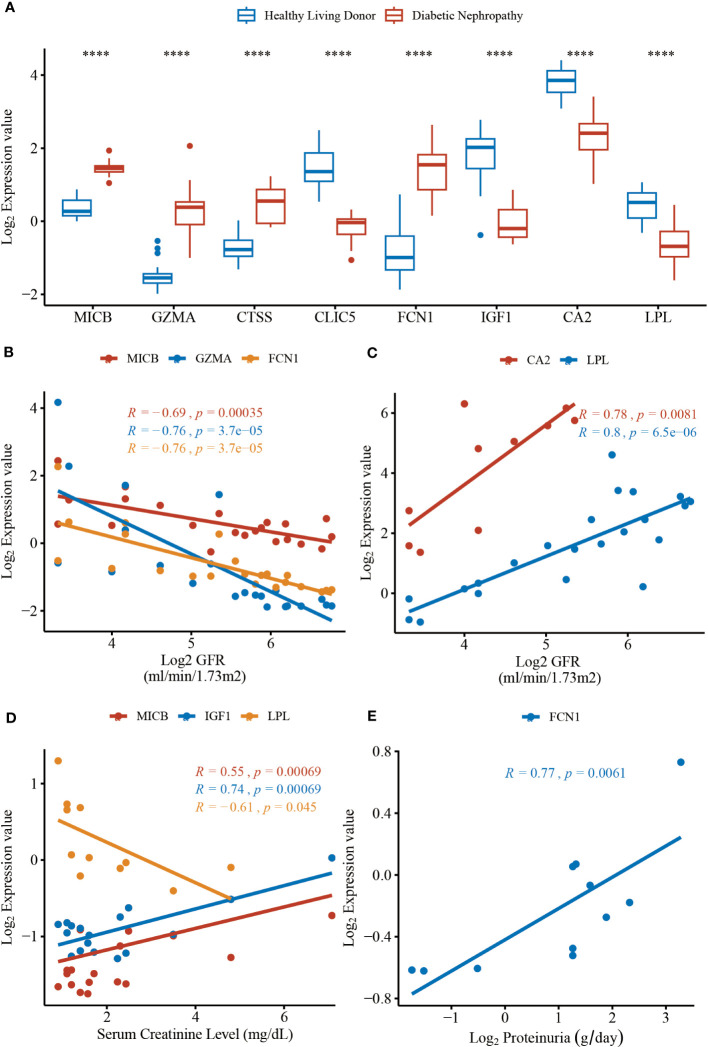
The mRNA expression levels of hub genes and clinical correlation from data in Nephroseq v5 online tool. **(A)** The expression level of *MICB* (*P* = 3.10 × 10^-12^), *GZMA* (*P* = 3.10 × 10^-6^), CTSS (*P* = 6.60 × 10^-7^), *CLIC5* (*P* = 9.50 × 10^-16^), FCN1 (*P* = 9.30 × 10^-9^), *IGF1* (*P* = 3.50 × 10^-9^), *CA2* (*P* = 5.10 × 10^-6^), *LPL* (*P* = 6.10 × 10^-7^). **(B)** The expression of MICB, GZMA, and FCN1 negatively correlated with GFR. **(C)** The expression of CA2 and LPL positively correlated with GFR. **(D)** The expression of MICB, IGF1 and LPL correlated with serum creatinine level. **(E)** The expression of LPL positively correlated with proteinuria. The DN groups represent the diabetic nephropathy patients and the Normal groups represent the healthy controls. GFR, glomerular filtration rate. ****P < 0.0001.

To validate the expression level of the identified protein in MR analysis, the Nephroseq v5 online tool was used. The results shown in **Figure 5A** indicated that the mRNA expression of *MICB*, *GZMA*, *CTSS*, and *FCN1* were significantly upregulated in the diabetic nephropathy glomerulus sample compared with the healthy controls. Meanwhile, the mRNA expression of *CLIC5*, *IGF1*, *CA2*, and *LPL* were significantly downregulated in glomerulus tissues of diabetic nephropathy patients. It is evident that the expression levels of 4 upregulated and 4 downregulated genes corresponded with the results we presented in the RRA analysis, which made the RRA results more persuasive and convincing. As for correlation with GFR level and gene expression illustrated in **Figures 5B, C**, a low level of GFR was significantly related to a high expression level of *MICB* (R = -0.69, P = 3.50 × 10^-4^), *GZMA* (R = -0.76, P = 3.70 × 10^-5^) and *FCN1* (R = -0.76, P = 3.70 × 10^-5^). Moreover, a high level of GFR was significantly correlated with a high expression level of *CA2* (R = 0.78, P = 8.10 × 10^-3^) and *LPL* (R = 0.80, P = 6.50 × 10^-6^). The mRNA expression level of *MICB* and *IGF1* manifested a positive correlation with serum creatinine in DN patients and the expression level of *LPL* reversely correlated with serum creatinine (**Figure 5D**). Besides, the mRNA expression level of *FCN1* in the renal glomerulus positively correlated with proteinuria (**Figure 5E**). The results shown in **Figure 5** indicate that *MICB*, *GZMA*, *FCN1*, and *IGF1* may be involved in promoting the development of diabetic nephropathy and *CA2* and *LPL* may play a protective role in the progression of diabetic nephropathy, which in accordance with the results of casual association from our MR analysis.

## 4 Discussion

DN is the leading cause of renal failure worldwide (23). Patients with renal complications caused by type 2 diabetes have a higher risk of death (24). Interactions between environmental and genetic factors promote the development of DN and related chronic kidney diseases (25). Glomerulopathy plays an essential role in the progression of DN (26). Proteinuria after decreased GFR is a clinical characteristic of DN (2). Renal pathological changes in DN first manifest as glomerular basement membrane thickening and mesangial expansion then progress to glomerular nodular lesions (27, 28). More genetic factors that may have an essential role in the progression of DN have been recently revealed. However, the mechanism of DN remains elusive and poorly understood which involves metabolic factors, oxidative stress, and renal hemodynamic (29, 30). Thus, further exploration of the underlying pathogenesis is urgently needed. Owing to the development of high-throughput microarray technology and progress in bioinformatics methods, we were able to detect potential hub genes that participate in the pathological mechanism and identify novel biomarkers of glomerular injury in DN. As a well-designed tool, the RRA algorithm is characterized by four key features: high computational efficiency, strong robustness to background noise, incomplete ranking, and significant scores for each element in the results (7). As a genetic epidemiological method, MR can overcome the limitations of traditional observational studies. To the best of our knowledge, our study is the first to systematically incorporate and integrate multiple microarray results to analyze the mechanism of glomerular injury in DN. In this study, we included five GEO datasets according to established criteria to identify DEGs from renal glomerular tissue samples between patients with DN and healthy controls. RRA analysis was employed to integrate DEGs from five GEO datasets with high statistical efficiency. Further functional annotation, protein-protein interaction network construction, and clinical validation were also performed to explore the potential roles of hub genes in DN.

In this study, to identify candidate biomarkers and verify the causal relationship between plasma proteins and DN, 82 DEGs, including 53 upregulated and 29 downregulated genes, were identified from multiple datasets through RRA analysis. A large-scale proteome MR analysis was also conducted. The results of the Gene Ontology (GO) and Kyoto Encyclopedia of Genes and Genomes (KEGG) enrichment analyses showed that the 82 DEGs were significantly enriched in neutrophil-related pathways (neutrophil degranulation, neutrophil activation involved in immune response, neutrophil activation, and neutrophil-mediated immunity). Michelis et al. (31) found that albumin modification and neutrophil activation participated in systemic inflammation and oxidative stress in DN. The KEGG pathways mainly included glycolysis/gluconeogenesis, steroid biosynthesis, tricarboxylic acid cycle, and pentose phosphate pathways. Glucose variability (GV)-related genes occupy central positions in networks of diabetic complications, such as DN (32). Subsequent MR analysis indicated the causal associations between MICB, GZMA, CTSS, CLIC5, FCN1, and DN risk; moreover, the associations for both MICB and GZMA were robust under multiple MR analysis methods. However, for CTSS and CLIC5, the direction of association with DN risk differed between our integrated multiple microarray and MR analyses, which requires further investigation. External validation and clinical correlations in the Nephroseq v5 online platform further confirmed that MICB, GZMA, FCN1, and IGF1 may promote the development and progression of DN, whereas CA2 and LPL have a protective effect against disease in patients with DN.

MICB, an immune-activating ligand for the killer cell lectin like receptor K1 (KLRK1)/NKG2D receptor, act as a stress-induced self-antigen recognized by gamma delta T cells (33). To date, no studies have clarified the association between MICB and DN. In our multiple microarray analysis, MICB was significantly upregulated in DN glomerulus samples, and MR analysis showed a robust causal association between higher genetically predicted levels of MICB and increased risk of DN. Moreover, higher MICB expression levels significantly correlated with worse renal function, including decreased GFR and elevated serum creatinine levels. Steinle et al. (34) found that MICB was involved in the immune response-activating cell surface receptor signaling pathway and can lead to cell lysis when bound to the KLRK1 receptor (35). A recent study found that the pattern of immune cell populations that infiltrate the tissue, such as neutrophils, lymphocytes, mast cells, and macrophages (36–38), plays an important role in the progression of DN, which is consistent with our enrichment findings on neutrophil-related pathways and immune responses. This could explain why MICB is elevated in patients with DN and related to the deterioration of renal function. As one of the immune receptors of MICB, NKG2D is mainly expressed in NK cells and distinct T-cell populations (39). A recent study found that several pathways related to immune, autophagy, and metabolic processes were significantly activated, including NK cell activation and resting NK cells in the glomerulus of DN (40), indicating that MICB may play a potential role by activating NKG2D on NK T cells in the progression of DN. Since there is still no research revealing the relationship between DN and MICB or its KLRK1/NKG2D receptor, future investigations are needed to explore how MICB participates in the pathogenesis of DN.

As an abundant protease in the cytosolic granules of cytotoxic T-cells and NK cells, GZMA can activate caspase-independent pyroptosis in target cells by catalyzing the cleavage of gasdermin B, leading to cell death (41). Wang et al. (42) revealed that the toll-like receptor 4/nuclear transcription factor κB signaling pathway could induce GSDMD-mediated pyroptosis in tubular cells in DN. Meanwhile, the activation of the NOD-like receptor thermal protein domain associated protein 3 could also induce pyroptosis in DN (43). In addition, several drugs targeting pyroptosis-associated proteins have been shown to have the potential to treat DN (44–46). In addition, GZMA also participates in the positive regulation of apoptotic processes and immune responses (47). These pathways were also included in our enrichment analysis of DEGs in DN. Our study showed that GZMA was upregulated in patients. Meanwhile, elevated levels of circulating GZMA were casually associated with a high risk of DN in the MR analysis and significantly correlated with a decreased GFR in the clinical aspect, suggesting that GZMA may be a promising therapeutic option for DN. However, this needs to be confirmed in future studies. Kummer et al. (48) found that no granzyme positive cells (cells that store GZMA and GZMB) were detected infiltrating tubular epithelium, and vascular and glomerular structures in renal biopsies from patients with various inflammatory, not transplant-related, renal diseases, indicating that the expression of GZMA in diabetic nephropathy might be specific.

Moreover, in our MR analysis, we found that increased CLIC5, FCN1, and IGF1 led to a higher risk of DN, whereas increased levels of CTSS, CA2, and LPL led to a lower risk of DN. Although the causal effects of FCN1, IGF1, CA2, and LPL were not significant owing to the lack of abundant genetic instruments, our clinical correlation provides some evidence of their roles in the progression of DN. A high level of FCN1 significantly correlated with a low GFR and increased proteinuria, while a high level of LPL significantly correlated with an increased GFR and decreased serum creatinine level. Nonetheless, these novel targets need to be confirmed in future studies. CLIC5 is required for the development and maintenance of proper glomerular endothelial cell and podocyte architecture (49) and has also been identified as a candidate biomarker for the diagnosis of DN through gene-based network analysis (50). CTSS is a thiol protease involved in the adaptive immune response and has been reportedly associated with the epithelial-mesenchymal transition of tubular epithelial cells in DN (51). FCN1 acts as a kind of extracellular lectin which functions as a pattern-recognition receptor in innate immunity (52) and is synthesized by peripheral leukocytes. A previous study found that FCN1 is associated with an earlier onset of type 1 diabetes in a cohort of children and adolescents. Moreover, FCN1 is differentially expressed in both DN and non-alcoholic fatty liver disease and is one of the ten optimal crosstalk genes in these two diseases selected by LASSO regression and Boruta algorithm (53). IGF1 was identified as an upregulated gene in DN and reported to be associated with renal hypertrophy and hyperfiltration in diabetic rats, which can be relieved by nitric oxide synthase inhibition (54). Brittain et al. (55) found the downregulation of renal IGF1 gene expression in several different chronic human kidney diseases, including diabetic nephropathy, which supports our differential analysis that IGF1 was downregulated and might link with a high risk of DN (OR = 1.16). To conclude, the differential expression of IGF1 might not be specific in diabetic nephropathy and further efforts need to be made. CA2 can catalyze the reversible hydration of carbon dioxide and has been reported to be related to type 2 diabetes mellitus (56). Meanwhile, no research has been conducted on DN and dysfunction of CA2 might lead to renal tubular acidosis. Level of anti-CA2 antibody can reflect renal (especially proximal renal tubular) and hematologic impairment (57). LPL is the most relevant crosstalk gene between non-alcoholic fatty liver disease and DN (53). LPL was reported to be expressed in mesangial cells, but not epithelial cells in glomeruli. Moreover, hyperlipidemia accelerates the progression of glomerular diseases and the addition of exogenous lipoprotein lipase to mesangial cells has been shown to lead to enhanced binding of lipoproteins to these cells (58) and Appel et al. (59) reported that the fabric acid derivative gemfibrozil inhibits adipose lipolysis and increases lipoprotein lipase activity thus decreasing LDL synthesis and accelerating its removal in proteinuric diseases. However, whether the causative effects of lipoprotein lipase in diabetic nephropathy are specific or not still needs more work to find out. Our study showed that CA2 and LPL may play protective roles in the progression of DN, whichrequires further investigation.

This study has several strengths, including the integrated multiple-microarray analysis using the RRA method, genetic instruments from recent large-scale genome-wide studies (9–15), and expression validation and clinical parameter correlation in the external database. Although encouraging results were obtained, several limitations need to be considered when interpreting our results. First, the multiple-microarray analysis examined the mRNA levels of genes in kidney glomerulus samples. While our MR analysis measures the circulating protein concerning DN, the relationship between mRNAs and proteins could be affected by differences in translational efficiency, protein degeneration, contextual confounds, and protein-level buffering (60). Some proteins are expressed locally and not secreted into the circulation, which partially explains why 57 DEGs failed to match the available pQTLs in our MR analysis. Moreover, this study did not examine all the plasma proteins related to DN. Second, the *cis*-pQTLs used in our analysis were mainly obtained through two platforms, Olink and Somascan, which were not able to detect different isoforms or protein modifications, but could exhibit high-throughput efficiency and high specificity when quantifying proteins on a large scale. Thus, future research combining different proteomic platforms is needed to confirm the association between microarrays and MR analyses (61, 62). Third, most pQTLs had only one or two instrumental variables available for each protein after selection, making it difficult to perform sensitivity and post-MR analyses. This can be addressed by using larger sample datasets in future studies. Finally, our samples were confined to European populations, and caution should be exercised when extending our results to other ethnicities. Thus, more studies considering other races are needed. Nonetheless, we believe that our MR results provide insights into the pathological development of DN. Biological experiments are still necessary to understand the complex biology of DN and illustrate its underlying mechanisms.

## 5 Conclusions

In summary, we provide a deeper insight by performing RRA analysis of the complicated molecular signature of glomerular injury in DN, followed by functional annotation and MR analyses to identify potential therapeutic targets, such as MICB, GZMA, CTSS, CLIC5, and FCN1. Moreover, through GO and KEGG enrichment analyses, we found that the DEGs were mostly enriched in neutrophil-related pathways and immune responses. We then validated the expression of genes and analyzed the association between gene expression and the clinical features of DN using the Nephroseq v5 online platform, showing that MICB, GZMA, FCN1, and IGF1 may be involved in the development of DN, whereas CA2 and LPL may play protective roles in DN. However, the mechanisms underlying glomerular injury in DN have not been fully elucidated, and further studies are needed to explore the functions of these therapeutic targets in DN.

## Data availability statement

The original contributions presented in the study are included in the article/**Supplementary Material**. Further inquiries can be directed to the corresponding author.

## Author contributions

CF, YG, and YS designed this research. CF did the data acquisition. YG conducted the statistical analysis. CF and YG wrote the first draft of the manuscript. YS revised the manuscript and give the final approval for the manuscript submission. All authors contributed to the article and approved the submitted version.
