# Supplementary material for: Integrated multiple-microarray analysis and mendelian randomization to identify novel targets involved in diabetic nephropathy
Source: Front Endocrinol (Lausanne). 2023 Jul 10;14:1191768. doi: 10.3389/fendo.2023.1191768 (PMC10363738; doi:10.3389/fendo.2023.1191768)
Supplement: Supplementary file 1 [file Image_1.pdf]

Supplementary Material

Integrated multiple-microarray analysis and mendelian randomization to identify novel targets involved in diabetic nephropathy

Supplementary Figures

Figure S1. Boxplots of the gene expression data in the five GEO datasets.

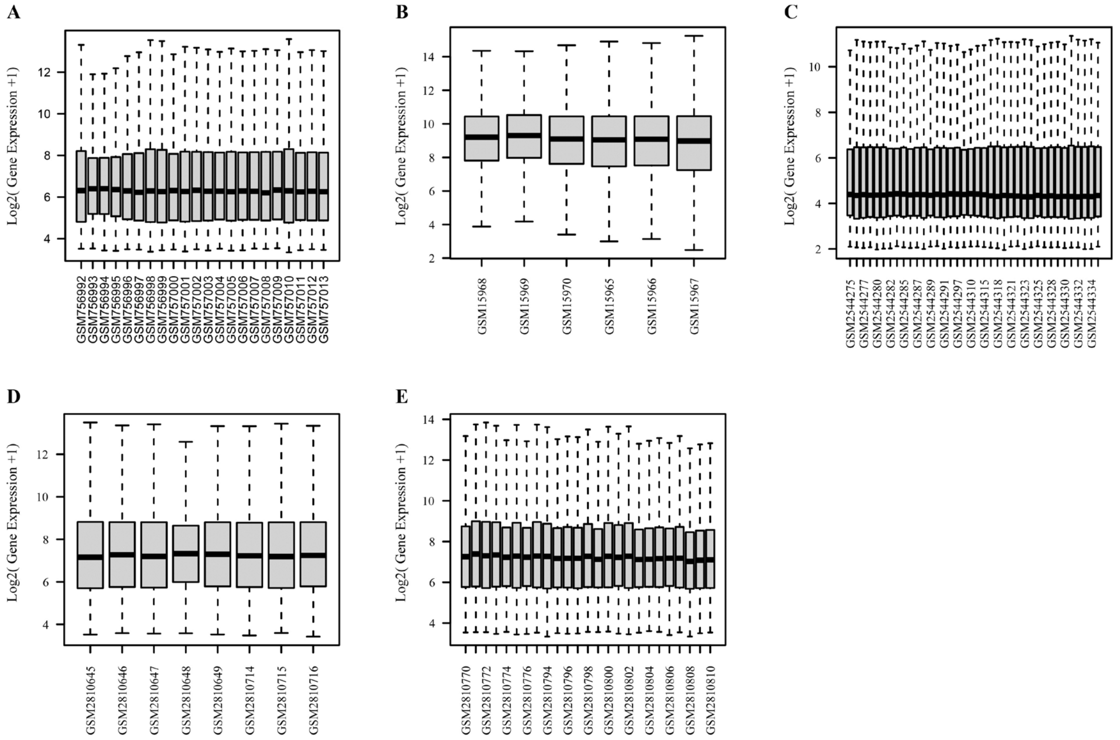

(A) GSE30528, (B) GSE1009, (C) GSE96804, (D) GSE104948_GPL24120, (E) GSE104948_GPL22945. The x-axis label represents the sample series and the y-axis represents the gene expression value. The horizontal black line in each box represents the median value of gene expression.

Figure S2. **PCA plots of the gene expression data in the five GEO datasets.**

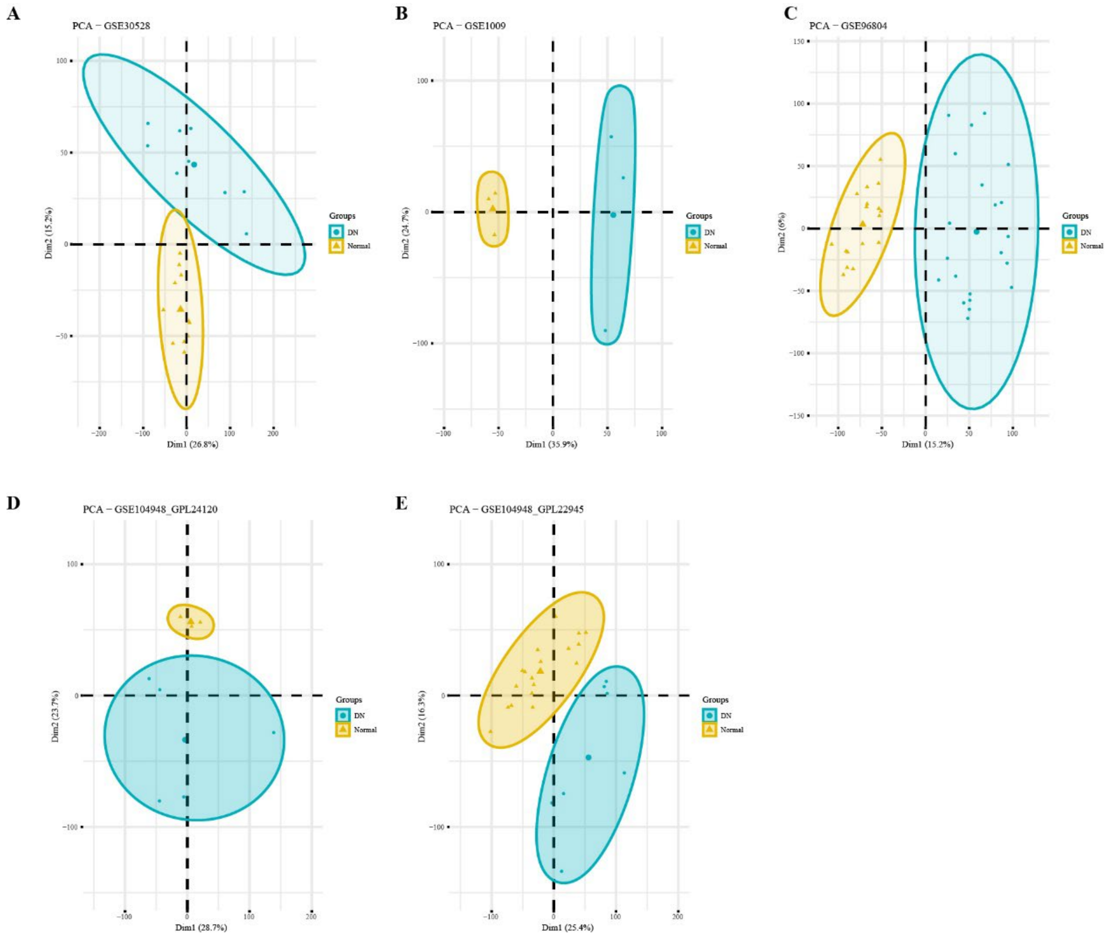

(A) GSE30528, (B) GSE1009, (C) GSE96804, (D) GSE104948_GPL24120, (E) GSE104948_GPL22945. The blue circle represents the diabetic nephropathy samples and the yellow circle represents the normal samples. The x-axis and the y-axis respectively describe two principal components.

Figure S3. Volcano plots of the gene expression data in the five GEO datasets.

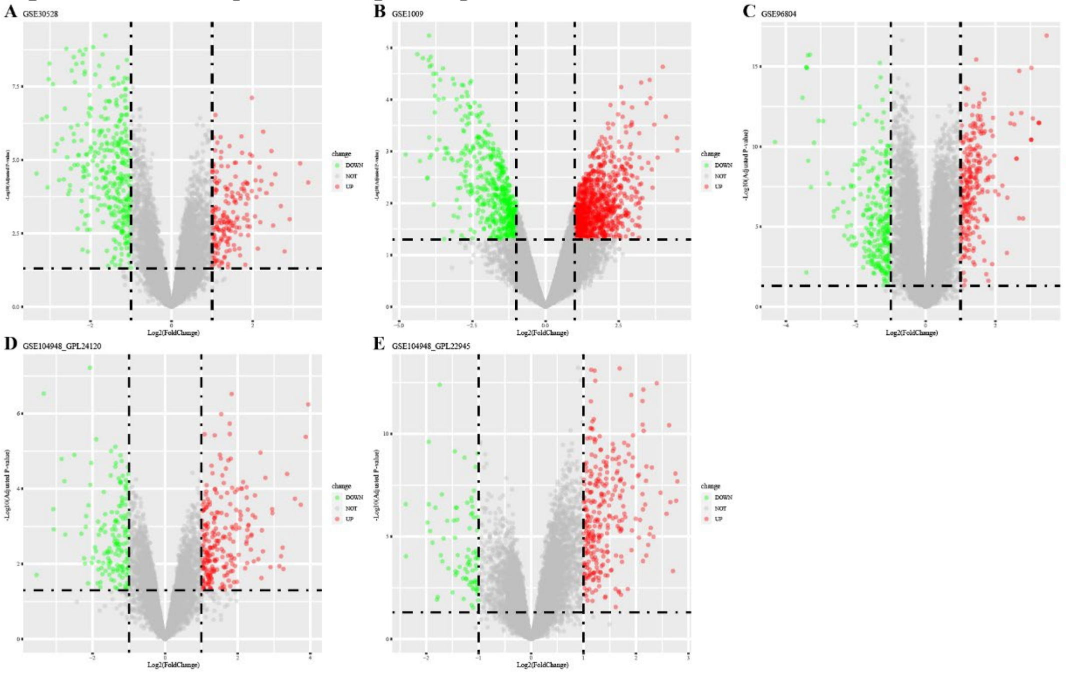

(A) GSE30528, (B) GSE1009, (C) GSE96804, (D) GSE104948_GPL24120, (E) GSE104948_GPL22945. The x-axis represents the log2(FoldChange) and the y-axis represents the -log10 (Adjusted P-value). The red dots indicate the upregulated genes and the green dots indicate the downregulated genes.
